# Synaptic footprints of time in working memory

**DOI:** 10.7554/eLife.110590

**Published:** 2026-02-16

**Authors:** Dhruv Grover, Marissa L Heintschel

**Affiliations:** 1 Kavli Institute for Brain and Mind, University of California, San Diego La Jolla United States; 2 Shu Chien-Gene Lay Department of Bioengineering, University of California, San Diego La Jolla United States

**Keywords:** computational neuroscience, working memory, temporal information, synaptic augmentation, None

## Abstract

Temporary changes in synapses may allow working memory to keep track of both events and their timing.

**Related research article** Mongillo G, Tsodyks M. 2025. Synaptic encoding of time in working memory. *eLife* **14**:RP107005. doi: 10.7554/eLife.107005.

From remembering a phone number just long enough to dial it, to keeping track of the steps in a recipe, we move through sequences of items and events without a second thought, instinctively aware of what comes next. Every step seems automatic, yet behind the scenes, our brains are performing a delicate balancing act, holding a few items in mind, while also keeping track of when each of them occurred. How does our “working memory” manage to do this?

Traditional working memory models focus on persistent neural activity or slow learning through repetition (the name Pavlov might ring a bell). These models can explain what information is stored, but they cannot explain how we can recall the order of items or the timing of events, especially for novel sequences (Buonomano and Maass, 2009; Goldman-Rakic, 1995). Yet behavioral research shows that people can immediately recall novel sequences, including their order and timing, without practice (Baddeley, 2003). This implies that working memory somehow stores temporal information in real time: this raises an obvious question – where is this information stored in the brain?

Now, in eLife, Gianluigi Mongillo and Misha Tsodyks propose that this temporal information is encoded directly in the dynamics of the synapses that connect neurons to other neurons (Mongillo and Tsodyks, 2025). Instead of storing temporal information separately, short-lived changes in synaptic signaling may embed a sense of time into memory itself.

Mongillo and Tsodyks extend their existing synaptic theory of working memory (Mongillo et al., 2008) by incorporating a process called synaptic augmentation: this is a type of short-term plasticity that develops slowly and persists for tens of seconds. Most neural signaling, on the other hand, is shaped by two forms of short-term plasticity – classical facilitation and depression – that typically occur on much shorter timescales (between milliseconds and a few seconds). The researchers propose that when a neuron fires repeatedly during a sequence, its outgoing synapses don’t just transmit the signal – they gradually increase in strength and retain a record of how recently and how often they were active. This forms a time-dependent gradient across synapses that effectively encodes when each item in a sequence occurred relative to others.

To illustrate, consider a sequence of memory items represented by different neural populations (Figure 1). As each item is presented, synaptic strengths in the corresponding neurons gradually increase. Because synaptic augmentation builds and decays slowly, the resulting pattern of synaptic strength retains a temporal fingerprint of the sequence – earlier items show stronger augmentation than later ones. Functionally, this temporal gradient allows our working memory to replay the sequence in the correct order and with approximate timing, at real speed or in time-compressed form that is faster.

**Figure 1.**
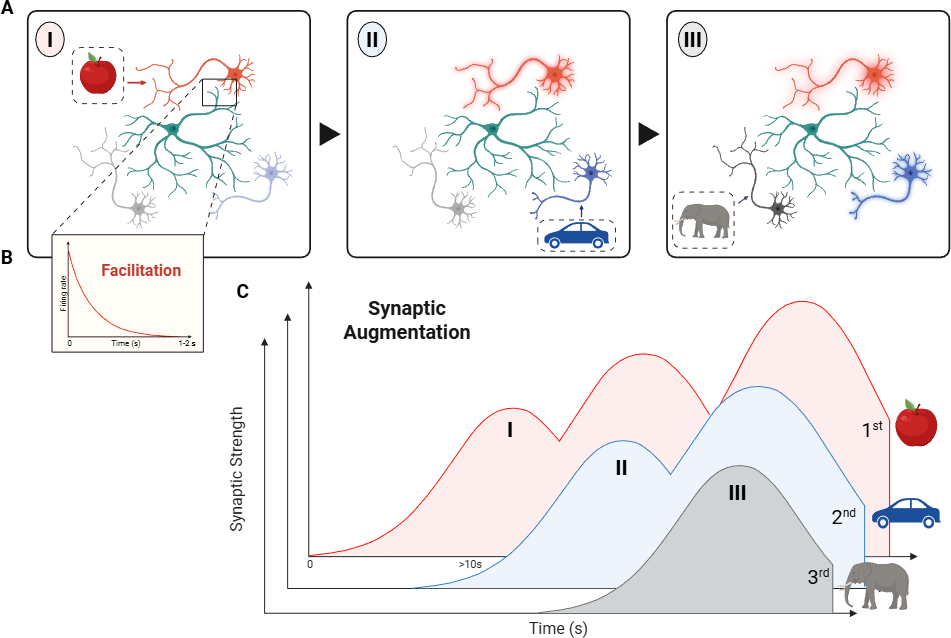
Schematic illustration of synaptic augmentation enabling the recall of temporal information. (**A**) When we see a sequence of objects – such as an apple (left) followed by a car (middle) followed by an elephant (right) – each object evokes a response from a specific subpopulation of excitatory neurons (represented here by the red, blue and gray neurons respectively). (**B**) The firing rate of each set of neurons increases rapidly, and then decays over a period of a few seconds: this is the classical facilitation and depression response that is traditionally associated with short-term plasticity. For clarity, the figure only shows this response for the red neurons that fire when we see the apple. Inhibitory neurons (represented here by the green neurons) are not directly activated by the objects, but they have a role in maintaining a neural representation of the objects. (**C**) However, this model is not able to explain how time is encoded in working memory. To address this challenge, Mongillo and Tsodyks propose that a form of short-term plasticity called synaptic augmentation is also involved. This process causes the synapses of the different subpopulations of neurons to increase and then decrease in strength more slowly than in the classical facilitation and depression response. Moreover, the overall strength of the synapses increases every time the neurons fire, so the synaptic strength for the first item (apple) is higher than that for the second item (car), which is higher than that for the third item (elephant), and so on. This gradient of synaptic strengths thus encodes the order in which the objects were seen and other temporal information.

This approach mirrors observations from experimental studies showing that activity during working memory is dynamic rather than static (Zylberberg and Strowbridge, 2017). Memory representations fluctuate in strength over time, can be briefly reactivated, and may be replayed during sleep or rest, sometimes in a time-compressed form that is thought to support learning and memory consolidation. Notably, Mongillo and Tsodyks show that without synaptic augmentation, the system quickly falls into a steady state where all past items are maintained equally, causing any temporal distinction between them to be lost. Augmentation, by contrast, preserves a gradient that naturally encodes order and elapsed time.

While the model proposed by Mongillo and Tsodyks – who are based at the Institute for Advanced Study in Princeton, Sorbonne University, CNRS, and the Weizmann Institute of Science – is conceptual rather than being based on specific neural recordings, it does offer testable predictions. The slow buildup and decay of synaptic augmentation align with behavioral observations showing that working memory can maintain temporal intervals of several seconds without active rehearsal (Fuster and Alexander, 1971). The theory further predicts that selectively disrupting augmentation mechanisms should impair memory in terms of the order and timing of items, while sparing memory for the items themselves.

Beyond behavior, the model provides a framework for interpreting neural activity patterns observed in electrophysiological studies. It accounts for ramping activity during memory delays, as well as “activity-silent” states where latent information can be reactivated by cues (Stokes, 2015). By linking these dynamic patterns to underlying synaptic changes, the model bridges the gap between observable neural activity and the synaptic process that underlies working memory.

In sum, the work of Mongillo and Tsodyks reframes a classic question in cognitive neuroscience: time is not an add-on to memory but an emergent property of how synapses change in real time (Kukushkin and Carew, 2017). The brain doesn’t need a separate “clock” to timestamp experiences: rather, it uses its own plasticity mechanisms to let time leave an imprint on memory. Models like this will continue to guide how we think about cognition – not as static repositories of information, but as active, changing processes shaped by brain rhythms and slow changes in the connections between cells (Miller et al., 2018).
